# Draft genome sequence of *Staphylococcus urealyticus* strain MUWRP0921, isolated from the urine of an adult female Ugandan

**DOI:** 10.1128/mra.00817-23

**Published:** 2023-12-11

**Authors:** Godfrey Wokorach, Bernard Erima, Florence Najjuka, James Kiyengo, Hannah Kibuuka, Ambrose K. Musinguzi, Fred Wabwire-Mangen, Denis K. Byarugaba

**Affiliations:** 1 Makerere University Walter Reed Project, Kampala, Uganda; 2 Multifunctional Research Laboratories, Gulu University, Gulu, Uganda; 3 Makerere University, Kampala, Uganda; 4 Uganda People's Defence Forces, Kampala, Uganda; Loyola University Chicago, Chicago, Illinois, USA

**Keywords:** *Staphylococcus urealyticus*, bacteria, Uganda, urinary tract infection, antibiotic resistance

## Abstract

*Staphylococcus urealyticus* bacteria are pathogenic among immune-compromised individuals. A strain (MUWRP0921) of *Staphylococcus urealyticus* with a genome of 2,708,354 bp was isolated from Uganda and carries genes that are associated with antibiotic resistance, including resistance to macrolides (*erm(C*) and *mph(C'*)), aminoglycosides (*aac(6")-aph(2"*)), tetracyclines (*tet(K*)), and trimethoprim (*dfrG*).

## ANNOUNCEMENT

*Staphylococcus urealyticus*, a coagulase-negative bacteria, causes infections in immunocompromised individuals (1). The bacteria are often linked to community infections, and some strains have developed resistance to multiple antibiotics (2, 3). Additionally, they can transfer antibiotic resistance genes to other pathogenic, nosocomial bacteria. We report the genome of *S. urealyticus* isolated from a female patient with urinary tract infection at Bwera General Hospital in Kasese district.

A swab dipped into 5 mL of void-collected urine was spread on blood agar, and resulting bacteria were sub-cultured on nutrient agar, Gram stained, and tentatively identified as coagulase-negative *Staphylococcus*. A culture from Luria broth was used for DNA extraction using DNeasy UltraClean Microbial Kit (Qiagen, Germantown, Maryland, USA). All culturing was done at 37°C for 24 hours. The Kapa HyperPlus Library Preparation Kit (Roche Diagnostics, Indianapolis, IN, USA) was used to create DNA libraries that were paired-end sequenced (2 × 300 bp) on Illumina NextSeq (Illumina, Inc., San Diego, CA) and yielded 2,659,332 (*R*1 = 1,329,666 and *R*2 = 1,329,666) sequence reads. Raw reads were examined using FastQC v0.11.9 (https://www.bioinformatics.babraham.ac.uk/projects/fastqc/) and trimmed using Btrim v0.2.0 (4). Sequence assembly was done using Newbler v2.7 and yielded coverage of 589× (5). CheckM v1.1.6 was used to determine the genome’s completeness (6). DFAST v1.2.20 was used to predict coding sequences, ribosomal RNAs (rRNAs), transfer RNAs (tRNAs), and CRISPR (7). Taxonomic assignment was done using Genome Taxonomy Database (GTDB Release 214.1) and Type (Strain) Genome Server by matching to the closest strain using an average nucleotide identity index (ANI) of ≥95% calculated using FastANI v1.1 (8 – 10). FASTME 2.1.6.1 was used to estimate a balanced minimum evolution tree with branch support using intergenomic distances and annotated in MEGA v11 (9, 11, 12). Resfinder v4.3.2 was used to predict antimicrobial resistance genes (13). PlasmidFinder-2.0 and blastn were used to confirm plasmid presence (14, 15). Unless otherwise stated, all software utilized default settings. The study was approved by the Makerere University School of Public Health Higher Degrees and Research Ethics Committee (HDREC 087) and Walter Reed Army Institute of Research IRB (WRAIR #1711).

**Fig 1:**
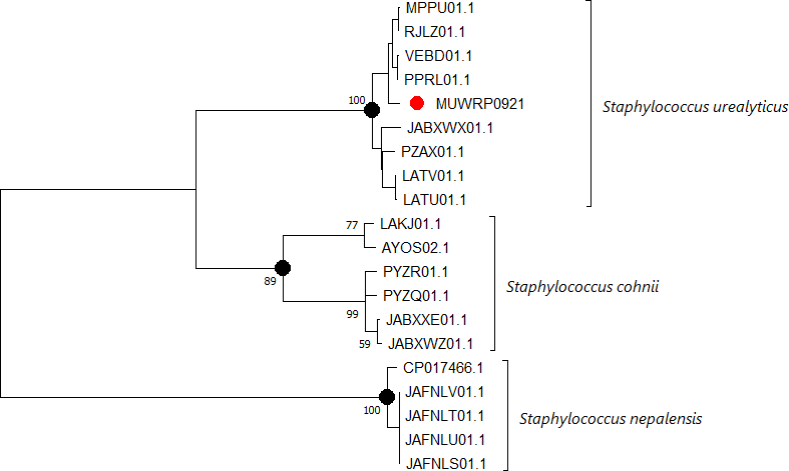
A tree was created using FastME 2.1.6.1 based on Genome BLAST Distance Phylogeny(GBDP) distances that were calculated from genome sequences. The branch lengths are proportional to the GBDP distance formula, and the numbers above each branch indicate GBDP bootstrap support values greater than 60% from 100 replications. The tree was rooted at the midpoint. The tree was uploaded to MEGA v11 for viewing and annotation. The red node shows strain MUWRP0921.

The assembly resulted in 29 contigs with an N50 value of 435,753. The genome completeness was 99.25%, and the genome size was 2,708,354 bp. The GC content was 32.5%, and 83.7% of the genome was predicted to be coding regions. The annotation predicted 2,672 coding sequences, 3 rRNAs, and 57 tRNAs to occur within the genome. Genome Taxonomy Database and Type Strains Genome Server concordantly identified strain MUWRP0921 as *Staphylococcus urealyticus*. Average nucleotide identity of strain MUWRP0921 with all the reference isolates for *Staphylococcus urealyticus* was ≥98%. Meanwhile, the ANI of strain MUWRP0921 with all *Staphylococcus cohnii* was ≤91.75%. Strain MUWRP0921 clustered together with strains of *Staphylococcus urealyticus* in a phylogenetic tree (Fig. 1). Strain MUWRP0921 carried *erm(C*), *dfrG*, *tet(K*), *aac(6′′)-aph(2′′),* and *mph(C*′) antibiotic resistance genes. Plasmid replicons (*rep10* and *rep7a*) were detected. *rep7a* Contig (4,409 bp) matches with *pML7056-2* (4,439 bp) and *p7GD01* (4,439 bp) plasmids with ≥99.95% identity/query cover. *rep10* Contig (2,473 bp) matches *pC344_1* (2,473 bp) and *pC247_1* (2,473 bp) with ≥99% identity/query cover.

## Data Availability

The whole-genome sequence of strain MUWRP0921 was submitted to NCBI and can be accessed with NCBI accession number JANBWB000000000. The version described in this paper is version JANBWB020000000. The raw sequences were deposited in the Sequence Read Archive (SRA) under the accession number SRR21141373.

## References

[1] Soldera J , Nedel WL , Cardoso PRC , d’Azevedo PA . 2013. Bacteremia due to Staphylococcus cohnii ssp. urealyticus caused by infected pressure ulcer: case report and review of the literature. Sao Paulo Med J 131:59–61. doi:10.1590/s1516-31802013000100010 23538597 PMC10852079

[2] Drozenová J , Petrás P . 2000. Characteristics of coagulase-negative staphylococci isolated from hemocultures. Epidemiol Mikrobiol Imunol 49:51–58. 10838776

[3] Lienen T , Schnitt A , Hammerl JA , Marino SF , Maurischat S , Tenhagen B-A . 2021. Multidrug-resistant Staphylococcus cohnii and Staphylococcus urealyticus isolates from German dairy farms exhibit resistance to beta-lactam antibiotics and divergent penicillin-binding proteins. Sci Rep 11:6075. doi:10.1038/s41598-021-85461-6 33727647 PMC7966787

[4] Kong Y . 2011. Btrim: a fast, lightweight adapter and quality trimming program for next-generation sequencing technologies. Genomics 98:152–153. doi:10.1016/j.ygeno.2011.05.009 21651976

[5] Silva GG , Dutilh BE , Matthews TD , Elkins K , Schmieder R , Dinsdale EA , Edwards RA . 2013. Combining de novo and reference-guided assembly with Scaffold_builder. Source Code Biol Med 8. doi:10.1186/1751-0473-8-23 PMC4177539 24267787

[6] Parks DH , Imelfort M , Skennerton CT , Hugenholtz P , Tyson GW . 2015. CheckM: assessing the quality of microbial genomes recovered from isolates, single cells, and metagenomes. Genome Res 25:1043–1055. doi:10.1101/gr.186072.114 25977477 PMC4484387

[7] Tanizawa Y , Fujisawa T , Kaminuma E , Nakamura Y , Arita M . 2016. DFAST and DAGA: web-based integrated genome annotation tools and resources. Biosci Microbiota Food Health 35:173–184. doi:10.12938/bmfh.16-003 27867804 PMC5107635

[8] Parks DH , Chuvochina M , Rinke C , Mussig AJ , Chaumeil PA , Hugenholtz P . 2022. GTDB: an ongoing census of bacterial and archaeal diversity through a phylogenetically consistent, rank normalized and complete genome-based Taxonomy. Nucleic Acids Res 50:D785–D794. doi:10.1093/nar/gkab776 34520557 PMC8728215

[9] Meier-Kolthoff JP , Carbasse JS , Peinado-Olarte RL , Göker M . 2022. TYGS and LPSN: a database tandem for fast and reliable genome-based classification and nomenclature of prokaryotes. Nucleic Acids Res 50:D801–D807. doi:10.1093/nar/gkab902 34634793 PMC8728197

[10] Jain C , Rodriguez-R LM , Phillippy AM , Konstantinidis KT , Aluru S . 2018. High throughput ANI analysis of 90K prokaryotic genomes reveals clear species boundaries. Nat Commun 9:5114. doi:10.1038/s41467-018-07641-9 30504855 PMC6269478

[11] Lefort V , Desper R , Gascuel O . 2015. FastME 2.0: a comprehensive, accurate, and fast distance-based phylogeny inference program. Mol Biol Evol 32:2798–2800. doi:10.1093/molbev/msv150 26130081 PMC4576710

[12] Tamura K , Stecher G , Kumar S . 2021. MEGA11: molecular evolutionary genetics analysis version 11. Mol Biol Evol 38:3022–3027. doi:10.1093/molbev/msab120 33892491 PMC8233496

[13] Bortolaia V , Kaas RS , Ruppe E , Roberts MC , Schwarz S , Cattoir V , Philippon A , Allesoe RL , Rebelo AR , Florensa AF , et al. . 2020. Resfinder 4.0 for predictions of phenotypes from genotypes. J Antimicrob Chemother 75:3491–3500. doi:10.1093/jac/dkaa345 32780112 PMC7662176

[14] Carattoli A , Zankari E , García-Fernández A , Voldby Larsen M , Lund O , Villa L , Møller Aarestrup F , Hasman H . 2014. In silico detection and typing of plasmids using plasmidfinder and plasmid multilocus sequence typing. Antimicrob Agents Chemother 58:3895–3903. doi:10.1128/AAC.02412-14 24777092 PMC4068535

[15] Camacho C , Coulouris G , Avagyan V , Ma N , Papadopoulos J , Bealer K , Madden TL . 2009. BLAST+: architecture and applications. BMC Bioinformatics 10. doi:10.1186/1471-2105-10-421 PMC2803857 20003500

